# Chitin Modulates Innate Immune Responses of Keratinocytes

**DOI:** 10.1371/journal.pone.0016594

**Published:** 2011-02-24

**Authors:** Barbara Koller, Alisa Sophie Müller-Wiefel, Rudolph Rupec, Hans Christian Korting, Thomas Ruzicka

**Affiliations:** Department of Dermatology and Allergy, Ludwig-Maximilian-University, Munich, Germany; Ludwig-Maximilian-University, Germany

## Abstract

**Background:**

Chitin, after cellulose the second most abundant polysaccharide in nature, is an essential component of exoskeletons of crabs, shrimps and insects and protects these organisms from harsh conditions in their environment. Unexpectedly, chitin has been found to activate innate immune cells and to elicit murine airway inflammation. The skin represents the outer barrier of the human host defense and is in frequent contact with chitin-bearing organisms, such as house-dust mites or flies. The effects of chitin on keratinocytes, however, are poorly understood.

**Methodology/Principal Findings:**

We hypothesized that chitin stimulates keratinocytes and thereby modulates the innate immune response of the skin. Here we show that chitin is bioactive on primary and immortalized keratinocytes by triggering production of pro-inflammatory cytokines and chemokines. Chitin stimulation further induced the expression of the Toll-like receptor (TLR) TLR4 on keratinocytes at mRNA and protein level. Chitin-induced effects were mainly abrogated when TLR2 was blocked, suggesting that TLR2 senses chitin on keratinocytes.

**Conclusions/Significance:**

We speculate that chitin-bearing organisms modulate the innate immune response towards pathogens by upregulating secretion of cytokines and chemokines and expression of MyD88-associated TLRs, two major components of innate immunity. The clinical relevance of this mechanism remains to be defined.

## Introduction

Chitin, after cellulose the second most abundant polysaccharide in nature, is an essential component of exoskeletons of insects and protects these organisms from the harsh conditions in their environment [1]–[3]. Although the relevance of chitin for humans is poorly understood, chitin exposition is relevant at host-pathogen interfaces, such as the lung, the gut and the skin. In mice, chitin airway challenge was found to activate the innate immune system [4] and chitin was found to modulate alveolar macrophage activation *in vitro* [5]. In contrast, the potential effect of chitin on the skin is incompletely understood. Since chitin is a component of several microorganisms that are known to trigger skin allergies, such as cockroaches, and house dust mites [6], [7], keratinocyte-chitin interactions may play a key role in the regulation of epidermal immunity of the skin.

The immune system recognizes pathogens via distinct pattern recognition receptors (PRRs), prototypically Toll-like receptors (TLRs) [8]. TLRs act as sensors of microbial pathogens and trigger downstream immune responses, aiming to eliminate the invading pathogen [9]. Therefore, TLRs represent key receptors at host-pathogen interfaces, such as the skin. Ten different human and twelve murine TLRs have been identified so far [8]. Immortalized keratinocytes have been described to express TLR1-5 and TLR10 at mRNA level [10], while primary keratinocytes were found to mainly express TLR1, TLR2, TLR3, TLR5 and TLR9 but not TLR4, TLR6, TLR7, TLR8, or TLR10 [11], [12]. Other studies reported TLR1, 2 and 5 [13] or TLR2 and TLR4 [14]–[16] being the predominant receptors expressed. When viewed in combination, previous studies provided evidence that keratinocytes express a variety of mainly anti-bacterial (MyD88-dependent) TLRs that may serve as microbial sensors and modulators of host-pathogen interactions. Therefore, the understanding of the regulation of TLRs in keratinocytes is essential for innate immunity of the skin.

We hypothesized that chitin exposure activates innate immune responses of keratinocytes by modulating chemokine secretion and TLR expression. To test this hypothesis we utilized both immortalized and primary human keratinocytes as modeling systems and analyzed the effect of chitin on cytokines and chemokines release and MyD88-associated TLR expression at mRNA and protein level. These studies demonstrate that chitin modulates epithelial immunity of the skin by upregulating cytokine and chemokine production and increasing TLR4 expression on keratinocytes.

## Methods

### Cell culture

The immortalized keratinocyte cell line HaCat or primary human keratinocytes (HEK cells) were cultivated similarly as described previously [10], [12]. In brief, HaCaT cells were cultivated in DMEM medium supplemented with 10% FCS, 1% Antibiotic-Mixture (Penicillin, Streptomycin, Neomycin) and 2 mM glutamine. Primary human keratinocytes (HEK cells) were cultivated in EpiLife MEPI500CA medium supplemented with EDGS, EpiLife defined growth supplement and gentamycin/amphotericin. For HEK cell culture, flasks were precoated with coating-matrix 1∶10. Unless otherwise indicated, primary keratinocytes and HaCaT cells at 80–90% confluency and a density of 3×10^4^ cells per well were stimulated for the indicated conditions prior to RT-PCR, FACS or ELISA. All cell culture products were from Gibco (Invitrogen/Cascade Biologics) unless stated otherwise. Chitin fragments (intermediate chitin fragments) were generated as described previously [17]. The chitin used was LPS free based on Limulus assays (below the limits of detection). Chitin fragments were incubated with cells for 48 hours at three different concentrations (C 500 = 0.22 mg/ml; C 1000 = 0.5 mg/ml and C 2000 = 2.0 mg/ml). These concentrations were chosen since (i) previously published studies used these concentrations [17] and (ii) initial experiments in our experimental setting prior to this study demonstrated that lower chitin concentrations were inert on keratinocytes, that is had no effect on chemokine secretion, whereas higher concentrations induced keratinocyte necrosis, as assessed by propidium iodide staining and LDH release.

### ELISA

The concentrations of CXCL8 (IL-8), Thymic stromal lymphopoietin (TSLP) and Interleukin 6 (IL-6) in the medium after 48 hours of medium or TLR ligand stimulation was quantitated in triplicates by enzyme-linked immunosorbent assay (ELISA; R&D, Wiesbaden, Germany).

### Q-PCR

RNA was isolated from HaCat cells or HEK cells using the High Pure RNA Isolation Kit (Roche). Total RNA was isolated according to the manufacturer's instructions, treated with DNase and immediately reverse transcribed by means of random hexamer primers (Roche) and Superscript II RT (Invitrogen, Life Technologies). Contamination with genomic DNA was controlled by cDNA synthesis reaction without reverse transcriptase. Expression levels of TLRs were quantified in triplicate by real-time quantitative RT-PCR (Q-PCR) with the use of SYBR green and the iCycler iQ detection system (Biorad, Hercules, CA, USA). Cycle threshold (Ct) values for genes of interest were normalized to β-actin and used to calculate the relative quantity of mRNA expression by the ΔΔCT method. β-actin was selected as normalizing gene, because it was stable under the *in vitro* conditions tested. A melting curve analysis was performed at the end of each run to rule out contamination with unspecific by-products that affect the quantitation of the PCR product. Primers are listed in table 1.

**Table 1:** Primers.

Gene	Forward	Reverse
β-actin	CTCCGTGGCCTTAGCTGTG	TTTGGAGTACGCTGGATAGCCT
TLR1	CTGGTATCTCAGGATGGTGTGC	TTGGAGTTCTTCTAAGGGTATGTTCC
TLR2	GGCCAGCAAATTACCTGTGTG	AGGCGGACATCCTGAACCT
TLR4	CTGCAATGGATCAAGGACCA	TTATCTGAAGGTGTTGCACATTCC
TLR5	TCGAGCCCCTACAAGGGAA	CACTGAGACTCTGCTATACAAGCTA
TLR9	TGGTGTTGAAGGACAGTTCTCTC	CACTCGGAGGTTTCCCAGC
NOD1	GAGCAAAGTCGTGGTCAACA	ACAGCACGAACTTGGAGTCA
NOD2	GCAACAGAGTGGGTGACGA	CACACTGCCAATGTTGTTCC

### FACS

Freshly obtained HaCat cells or HEK cells were incubated with the respective monoclonal antibodies for 40 min, washed three times and analyzed by flow cytometry (FACSCalibur, Becton-Dickinson, Heidelberg, Germany). Ten thousand cells were analyzed per sample. Propidium iodide (PI, 5 µg/ml; Sigma, St. Louis, MO, USA) and Annexin V-FITC (5 µg/ml; Boehringer Mannheim, Mannheim, Germany) were used to exclude apoptotic (Annexin V^+^, PI^−^) and necrotic (Annexin V^+^, PI^+^) leukocytes. Only viable cells were included in the analysis. The following labelled monoclonal anti-human antibodies were from eBioscience (San Diego, CA, USA): mouse IgG_1_ TLR1-PE, mouse IgG_2a_ TLR2-fluorescein isothiocyanate (FITC), mouse IgG_1_ TLR4-PE and rat IgG_2a_ TLR9-PE. Mouse IgG_2a_ TLR5-PE was from Imgenex (San Diego, CA, USA). The following labelled monoclonal anti-human antibodies were from BD Pharmingen (San Diego, CA, USA), mouse IgG_1_-APC, mouse IgG_1_-PE, mouse IgG_2a_-PE, mouse IgG_2a_-FITC and rat IgG_2a_-PE. For TLR9 detection, permeabilized cells and intracellular staining techniques were used. Isotype controls were subtracted from the respective specific antibody expression and the results were reported as mean fluorescence intensity (MFI). Calculations were performed with Cell Quest analysis software (Becton-Dickinson, Heidelberg, Germany).

### Functional assays

To assess the functional relevance of TLR4 on HEK cells, lipopolysaccharide (LPS, Sigma Aldrich) was used at 100 ng/ml to stimulate cultured keratinocytes. A mouse anti-human TLR2 antibodies with neutralizing/blocking characteristics (Abcam) was used to assess whether chitin binds through TLR2 at keratinocytes.

### Statistics

Data are shown as means ± standard error of the mean (SEM). Comparisons among all groups were performed with ANOVA and comparisons between two groups were performed with the two-sided t test as described previously [18]. Correlation analysis was performed by calculating the two-tailed Pearson correlation coefficient. A *P* value of <0.05 was considered to be significant. A correlation was assumed when the correlation coefficient was >0.3. Statistical analysis was performed with Prism 4.0 (Graph Pad Software, San Diego, CA, USA) and STATA version 8.2 for Windows (STATA Corporation, College Station, TX, USA).

## Results

### Chitin is bioactive on keratinocytes and triggers cytokine and chemokine release

**Figure 1:**
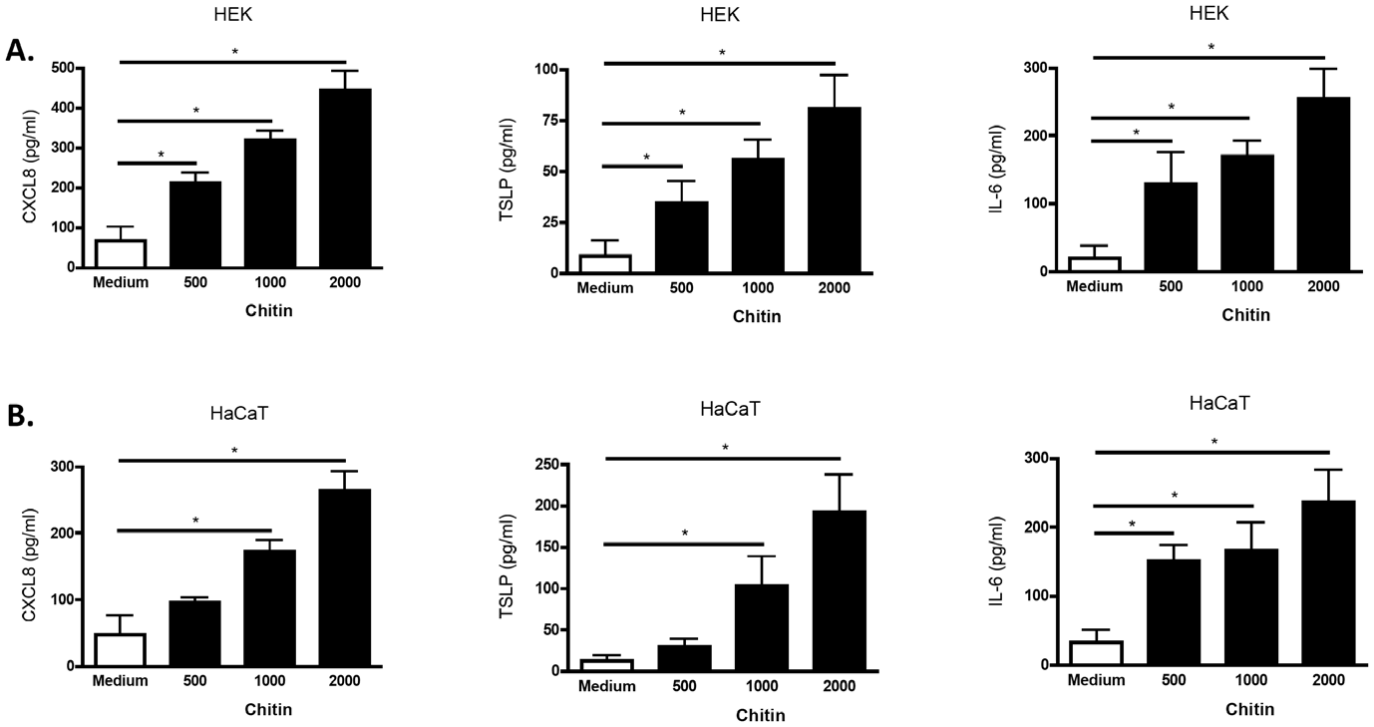
CXCL8 secretion. Figure A shows primary keratinocytes, Figure B immortalized HaCaT cells. CXCL8, TSLP and IL-6 secretion was measured in triplicates in supernatants after 48 h in medium or chitin-treated cells using ELISA. Chitin fragments were incubated with cells for 48 hours at three different concentrations (C 500 = 0.22 mg/ml; C 1000 = 0.5 mg/ml and C 2000 = 2.0 mg/ml). * p<0.05 of medium compared to chitin treated cells.

First, we stimulated immortalized and primary keratinocytes with different concentrations of chitin fragments and analyzed whether keratinocytes responded with secretion of a prototypical innate immune chemokine, CXCL8 (IL-8), known to be released by activated keratinocytes and recruiting neutrophils to the site of infection. Our studies demonstrated that chitin dose-dependently induced CXCL8 protein production by both primary (Figure 1A) and immortalized (Figure 1B) keratinocytes, with a stronger effect seen on primary (HEK) cells. This effect was not specific for CXCL8, since also other pro-inflammatory mediators (IL-6, TSLP) were induced by chitin stimulation (Figure 1A and B). Further studies showed that chitin had a distinct effective concentration range of pro-inflammatory bioactivity, as higher chitin concentrations induced keratinocyte cell necrosis (LDH release and propidium iodide staining), whereas lower concentrations had no bioactive effect and were inert (data not shown). These studies demonstrate that chitin fragments are bioactive on keratinocytes by inducing cytokine and chemokine production and suggest that skin contact with chitin-bearing microorganisms could act pro-inflammatory by triggering neutrophilic inflammation.

Previous studies indicated that chitin is sensed through TLR2 [17]. Therefore, we blocked TLR2 on keratinocytes using antibodies prior to chitin treatment and found that the chitin-induced effects were largely abrogated when TLR2 was blocked (Table S1).

### Chitin upregulates TLR and NOD gene expression in keratinocytes

**Figure 2:**
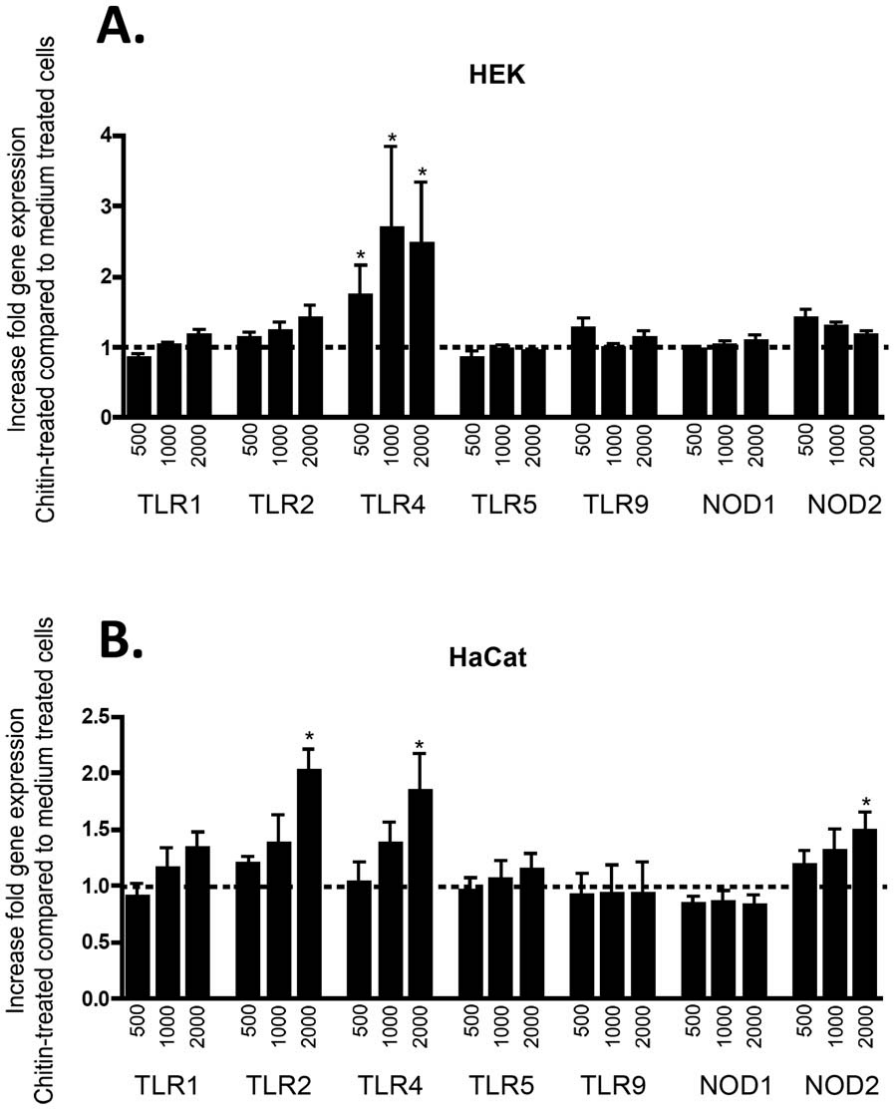
Q-PCR results. Figure A shows primary keratinocytes, Figure B immortalized HaCaT cells. Relative gene expression was analyzed using quantitative real-time RT-PCT (Q-PCR) and was normalized to β-actin as housekeeping gene. Chitin fragments were incubated with cells for 48 hours at three different concentrations (C 500 = 0.22 mg/ml; C 1000 = 0.5 mg/ml and C 2000 = 2.0 mg/ml). Shown is the fold increase of relative gene expression chitin compared to medium treated cells. * p<0.05 of medium compared to chitin treated cells.

TLRs modulate the innate immunity toward pathogen and danger-associated molecular pattern by promoting production of pro-inflammatory chemokines. Therefore, we tested whether the chitin-induced cytokine and chemokine secretion was associated with a modulation of TLR expression pattern by keratinocytes. In primary keratinocytes, chitin significantly upregulated TLR4 mRNA expression, whereas other TLR or NOD gene expression levels were unaffected (Figure 2A). Consistently in HaCaT cells, chitin increased TLR4 mRNA expression dose-dependently, but also enhanced gene expression of TLR2 and, to a lesser extent, the non-TLR PRR NOD2 (Figure 2B). Increases in TLR4 mRNA expression levels correlated positively with increases in TLR4 protein expression levels for individual experiments (r = 0.87, * p<0.05). These studies demonstrate that chitin modulates gene expression of pattern recognition receptors, in particular TLR4.

### Chitin upregulates TLR4 protein expression in keratinocytes

**Figure 3:**
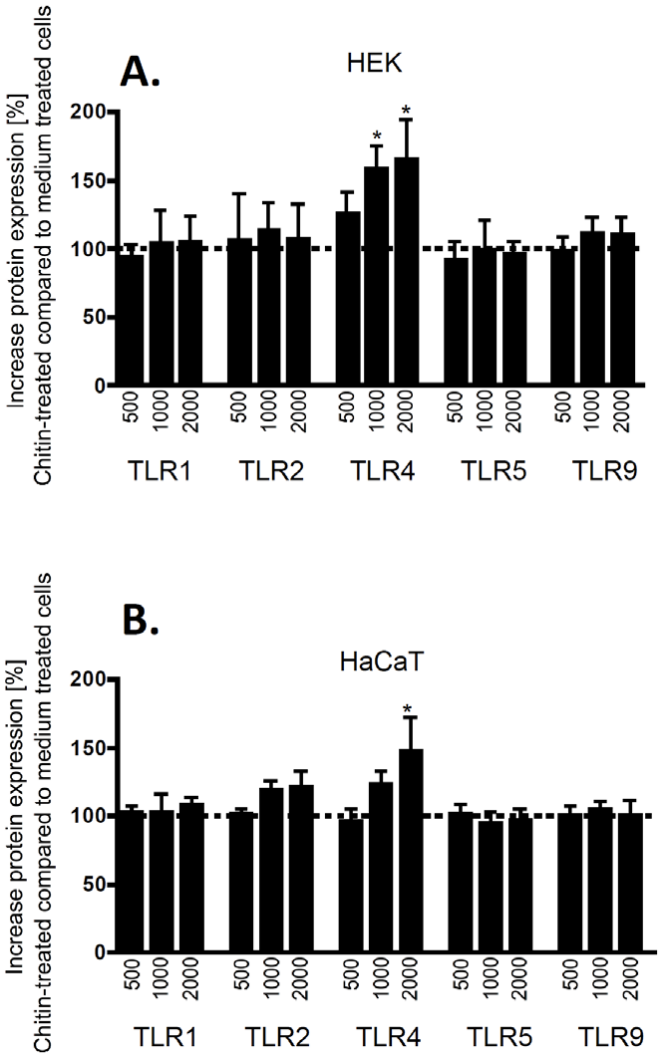
FACS results. Figure A shows primary keratinocytes, Figure B immortalized HaCaT cells. Chitin fragments were incubated with cells for 48 hours at three different concentrations (C 500 = 0.22 mg/ml; C 1000 = 0.5 mg/ml and C 2000 = 2.0 mg/ml). Shown is the % increase of TLR surface (TLR1, TLR2, TLR4, TLR5) or intracellular (TLR9, NOD1, NOD2) expression of chitin treated cells compared to medium treated cells. * p<0.05 of medium compared to chitin treated cells.

**Figure 4:**
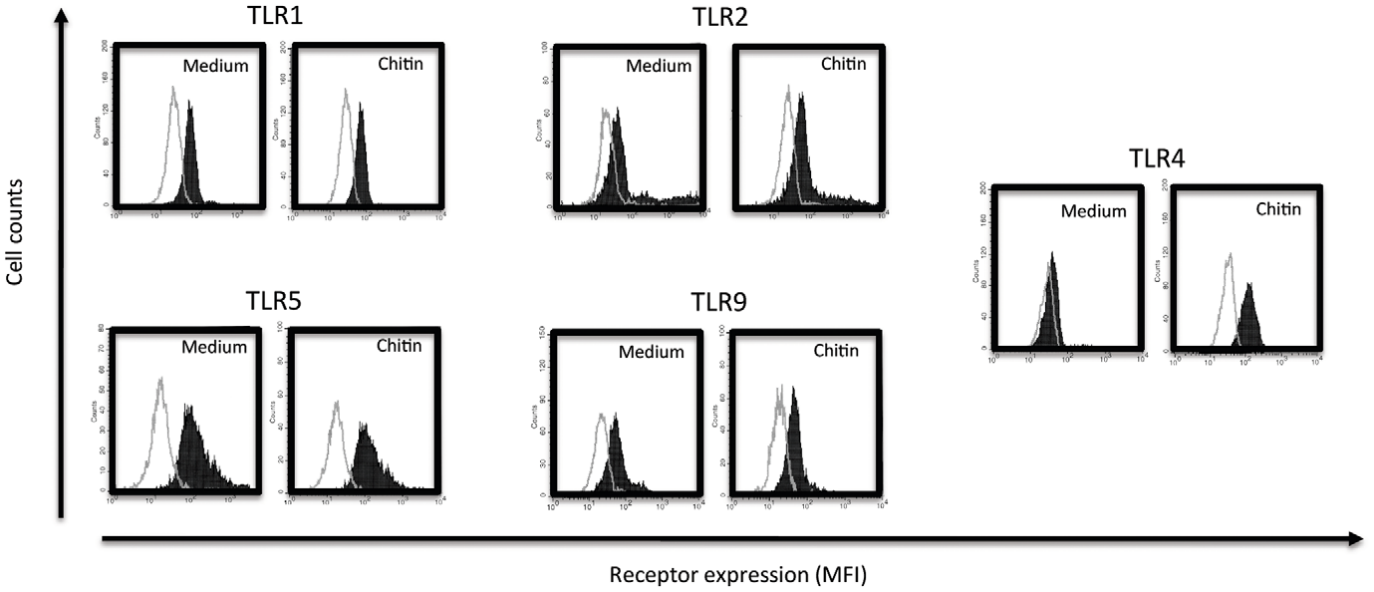
Representative FACS result. Representative FACS histograms of medium and chitin treated primary keratinocytes (HEK cells). MFI: mean fluorescence intensity. The unfilled histograms represents the respective isotype controls, the filled histograms the specific antibody stainings for medium- or chitin-treated cells. Note: chitin treatment upregulates TLR4 surface expression on keratinocytes.

To investigate whether chitin-induced modulation of mRNA expression is also reflected by protein expression changes, we quantified TLR and NOD protein expression using flow cytometry. In primary keratinocytes, chitin treatment dose-dependently upregulated TLR4 surface expression, while chitin had no significant effects on other TLR receptors (Figure 3A and Figure 4). Similarly in HaCat cells, chitin dose-dependently upregulated TLR4 expression without modulating other TLR receptors. Comparing intracellular (cytosolic) and membranous (surface) receptor pools, we found that chitin-induced TLR4 upregulation was not due to increased translocation from intracellular receptor storage pools (data not shown), suggesting that chitin mediated its effects at the transcriptional level. These studies confirmed our gene expression results and show that chitin modulates innate immune pathways by upregulation of TLR4 protein expression. To test whether the upregulation of TLR4 protein on keratinocytes had any functional relevance, we stimulated keratinocytes after chitin treatment with the TLR4 ligand LPS and quantified chemokines release as functional read-out. These studies demonstrated that LPS triggered chemokine release after chitin-induced TLR4 upregulation, but not without prior chitin priming (Table S1). When viewed in combination, these studies indicate that chitin is sensed through TLR2 and induces chemokine release and TLR4 expression by keratinocytes.

## Discussion

The skin represents the body's interface between the outer environment and epithelial innate immunity, where intimate interactions between keratinocytes and microorganisms take place. Chitin is an ubiquitous environmental polysaccharide. Both insects and allergen-inducing microorganisms, such as house-dust mites or *Aspergillus* fungi, contain chitin [2], [3], [19]. Traditionally, chitin has been assumed to have no effect on human immune responses, but recent studies challenged this notion by demonstrating that chitin fragments act as a pro-inflammatory stimulus on macrophages [4].

We hypothesized that chitin fragments stimulate keratinocytes and thereby modulates the innate immune response of the skin. Here we show that chitin is bioactive on primary and immortalized keratinocytes by triggering production of pro-inflammatory cytokines and chemokines. Paralleled with cytokine/chemokine induction, chitin stimulation upregulated the expression of TLR4 on primary and immortalized keratinocytes at mRNA and protein level and enabled functional responses of keratinocytes towards TLR4 ligands. Based on these findings, we speculate that chitin-bearing organisms modulate the innate immune response towards pathogens by upregulating production of chemokines and by increasing TLR4 surface expression. Accordingly, chitin contact may prime neutrophilic inflammation and thereby boost innate immunity against Gram-negative pathogens.

Despite previously described anti-viral and anti-tumor activities of chitin derivatives, such as chitosan, limited data is available regarding the immunological effects of chitin fragments that are generated at sites of infection and inflammation [1], [4], [19], [20]. Recently, studies demonstrated that chitin fragments modulate innate and adaptive immune responses by activating innate immune cells and inducing cytokine and chemokine production through distinct pattern recognition cell surface receptors in particular macrophage mannose receptor, TLR-2 and/or Dectin-1 [1], [4], [17], [19]. Based on these previous findings that chitin might be sensed through TLR2, we blocked TLR2 and found that the chitin-mediated effects were largely abrogated. Several studies analyzed the effect of chitin on macrophages *in vitro* [17], [21] and *in vivo* [4], [22] and showed that chitin stimulated macrophage IL-17A production and upregulated IL-17A receptor expression [17]. These studies further demonstrated that these effects were TLR-2 and MyD88-dependent. Additionally, these investigations demonstrated that IL-17A pathway activation was essential for some chitin-induced effects. Administration of chitin particles triggered activation of alveolar macrophages and triggered the expression of IL-12, tumor necrosis factor (TNF)-α, and IL-18 [5]. The latter studies also provided evidence that the chitin-induced effects on cytokine productions were mediated by a macrophage mannose-receptor (MMR) dependent phagocytic mechanism [23].

Recently, Reese *et al*. investigated the *in vivo* immune effects of chitin in airway inflammation [4]. In these studies, chitin coated beads administered into the airways of mice induced the accumulation of IL-4 expressing cells and the authors attributed these cells to eosinophils by using distinct cell surface markers, in particular siglec F+IL-4+ for eosinophils and basophils IgE+cKit−IL-4+. Furthermore, chitin treatment triggered alternative macrophage activation. This study suggested that chitin is involved in the pathogenesis of allergic/Th2 responses. This latter notion is challenged by studies that orally administered chitin inhibited allergen-induced IgE production [22]. Instillation of chitin micro-particles into the airways significantly down-regulated allergic responses to *Dermatophagoids pteronyssinus* (Der p) and *Aspergillus fumigatus* including IgE levels, IL-4 production, eosinophilia, airway hyper-responsiveness, and lung inflammation [24].

In contrast to the evidence of chitin in macrophage activation and Th2 immune responses in the airways, the potential effect of chitin on keratinocytes has not been defined so far. However, chitin contact may play a critical role in skin immunity since allergens such as house dust mites, fungi or insects contain chitin that is sensed by the epidermal cell layer through PRRs [1], [19]. Therefore, we studied the effects of chitin fragments on keratinocyte cytokine/chemokine secretion and TLR expression, two major components of innate immunity. These studies demonstrated that chitin dose-dependently upregulated secretion of CXCL8, a potent chemoattractant for neutrophils, that promotes bacterial clearance at sites of infection [25]. Accordingly, our studies imply that contact with chitin-bearing microbes could induce CXCL8 secretion at host-pathogen contact sites. The immunological consequences of CXCL8 increase at sites of chitin-skin interactions, however, could be two-faced: On the one hand, increased CXCL8 levels could feed the chemotactic gradient from skin-blood-bone marrow, thereby lowering the threshold for neutrophil recruitment upon later bacterial infections; on the other hand, chronic chitin stimulation may pave the way for the establishment and maintenance of auto-inflammatory skin inflammation. The duration of pathogen contact and the amount of microbial chitin required to elicit these responses remains to be defined. The hypothesis that chitin contact may favor neutrophilic inflammation is supported by the observation that epicutaneous sensitization with a chitin-bearing dust mite allergen resulted in localized dermatitis characterized by pronounced infiltration of neutrophils [26]. Nevertheless, the precise association between chitin exposure and CXCL8/neutrophilic inflammations remains to be characterized in future studies. Besides CXCL8, we found that chitin triggered increased production of TSLP by keratinocytes. The cytokine TSLP has been involved in the pathogenesis of allergic Th2-driven diseases and triggers the release of the chemokines CCL17 and CCL22. Based on our finding that chitin upregulated TSLP production we speculate that chitin – skin interaction modulates both neutrophilic as well as Th2-associated immune mechanisms.

Paralleled by the increase in CXCL8 secretion, we found that chitin modulated the innate immune sensing system of the skin. Chitin upregulated the LPS receptor TLR4 dose-dependently. This effect was consistent at RNA and protein level for both primary and immortalized keratinocytes, whereas for other TLR and non-TLR (NODs) receptors, the effects of chitin were either low or not consistent. The upregulation of TLR4 protein was functionally relevant since upregulated TLR4 receptors enabled LPS responsiveness by keratinocytes. These results tempt us to speculate that chitin exposure may shape the recognition of Gram negative pathogens, such as *Pseudomonas aeruginosa*, an opportunistic pathogen, commonly found in ulcerous skin lesions of the skin and sensed through MyD88-dependent pathways [27]-[31]. By upregulating TLR4, chitin may enhance innate immunity against Gram negative pathogens and may, in concert with CXCL8-mediated neutrophil recruitment, boost neutrophilic innate host defence in bacterial skin infections. This mechanisms may also have relevance for fungi, since human epithelial cells were reported to establish direct antifungal defense through TLR4-mediated signalling, a mechanism that involved neutrophilic inflammation [32]. Besides pathogen-associated molecular patterns (PAMPs), TLR4 has been reported to recognize also damage (host)-associated molecular patterns (DAMPs) [8]. Thus, the chitin-mediated effects could have an impact on disease conditions characterized by extracellular matrix break-down and tissue remodelling beyond infection.

In summary, we found that chitin is bioactive on keratinocytes by triggering production of pro-inflammatory cytokines and chemokines and by upregulating the expression of TLR4 on primary and immortalized keratinocytes at mRNA and protein level. We speculate that chitin-bearing organisms modulate the innate immune response towards pathogens and should be regarded as modulators of innate immunity of the skin.

## Supporting Information

Table S1 Data is shown for HEK cells. LPS was used at 100 ng/ml; Chitin at 2 mg/ml, Anti-TLR2 blocking antibodies (Abcam) at 20 µg/ml. CXCL8 levels were quantified by ELISA. *p<0.05 compared to medium.
(DOC)
