# Supplementary material for: Chitin Modulates Innate Immune Responses of Keratinocytes
Source: PLoS One. 2011 Feb 24;6(2):e16594. doi: 10.1371/journal.pone.0016594 (PMC3044707; doi:10.1371/journal.pone.0016594)
Supplement: Table S1 — Data is shown for HEK cells. LPS was used at 100 ng/ml; Chitin at 2 mg/ml, Anti-TLR2 blocking antibodies (Abcam) at 20 µg/ml. CXCL8 levels were quantified by ELISA. *p<0.05 compared to medium. (DOC) [file pone.0016594.s001.doc]

**Table S1**

Data is shown for HEK cells. LPS was used at 100ng/ml; Chitin at 2mg/ml, Anti-TLR2 blocking antibodies (Abcam) at 20μg/ml. CXCL8 levels were quantified by ELISA. **p<0.05 compared to medium*

| *Read-out* | *CXCL8 release* |
| --- | --- |
| Medium | 82±13 |
| LPS | 84±27 |
| Chitin | 450±59*** |
| 1. Chitin 2. LPS | 749±83*** |
| Anti-TLR2 | 85±17 |
| Anti-TLR2 + Chitin | 103±32 |
